# Evaluation of Quality of Life, Anxiety, and Depression in Patients with Primary Axillary Hyperhidrosis Undergoing Treatment with a Microwave Device: One-year Follow-up

**DOI:** 10.2340/actadv.v104.40543

**Published:** 2024-09-05

**Authors:** Maria PISSA, Rasha HASHEM, Alexander SHAYESTEH, Sarah WRISLEY, Emanuela MICU

**Affiliations:** 1 Department of Dermatology and Venereology, Ryhov County Hospital, Jönköping; 2 Department of Dermatology and Venereology in Östergötland, and Department of Biomedical and Clinical Sciences, Linköping University, Linköping; 3 Department of Public Health and Clinical Medicine, Dermatology and Venereology, Umeå University, Umeå; 4 Faculty of Medicine and Health Sciences, Linköping University, Sweden

**Keywords:** axillary, hyperhidrosis, microwave, quality of life

## Abstract

Hyperhidrosis is associated with social and emotional stress, affecting quality of life. Microwave energy technology treats primary axillary hyperhidrosis by thermolysis of sweat glands. The successful reduction of sweating in patients with primary axillary hyperhidrosis after microwave treatment has been studied, but there is limited evidence on the psychological and long-term effects. This study examined patient- reported outcome measures including depression and anxiety in patients with primary axillary hyperhidrosis and the effect of microwave therapy on these para-meters. Patients received 1 or 2 microwave-based treatments, within 3-month intervals. All patients were finally examined at approximately 1 year after the first treatment using the Hyperhidrosis Disease Severity Scale, Hyperhidrosis Quality of Life^©^, Dermatology Life Quality Index, and Hospital Anxiety and Depression Scale^©^. A total of 103 individuals with primary axillary hyperhidrosis were included in the study, with a Hyperhidrosis Disease Severity Scale score of 3 or 4. Statistically significant improvement in quality of life, anxiety, and depression scores were observed at 1-year follow-up. The primary endpoint, Hyperhidrosis Disease Severity Scale of 2 or less 1 year after the first treatment, was achieved by 88.2% of patients. No serious adverse side effects were observed.

## SIGNIFICANCE

People with primary axillary hyperhidrosis experience reduced quality of life. In this study, we wanted to investigate microwave treatment for severe primary axillary hyperhidrosis defined as a score of 3 or 4 on the Hyperhidrosis Disease Severity Scale. We included approximately 100 patients and after 1 year 88.2% of patients who received 1 or 2 microwave treatments achieved a Hyperhidrosis Disease Severity Scale score of 2 or less. Moreover, treat-ment has significantly improved quality of life, anxiety, and depression symptoms. This study provides further evidence on the effect microwave treatment has significantly improved quality of life, anxiety, and depression symptoms. This study provides further evidence on the effect microwave treatment has in primary axillary hyperhidrosis and highlights the burden of excessive sweating in daily and psychosocial life.

Primary localized hyperhidrosis is a chronic disorder of excessive sweat production without an underlying cause (1). It affects approximately 1% of the population, although the prevalence is higher in some countries, such as Sweden at 5.5% (2, 3). The condition is usually ongoing and may be confined to the axillae; however, is often multifocal and may affect the palms, soles, or face. It causes major problems in many social situations and has a negative impact on quality of life (QoL) by interfering with daily activities and causing anxiety and embarrassment (4–6).

Several studies have shown that anxiety, sleep disturbances, daytime tiredness, and depression are commonly associated with hyperhidrosis (7, 8). Although causality is difficult to assess, far fewer studies have been conducted on the effects of hyperhidrosis treatment on these psychological parameters (9).

Although most available treatments have only a time-limited effect, there are newer approaches that can provide long-lasting relief, such as fractionated microneedle radiofrequency (10) or microwaves (11–13). The mere thought of a permanent cure can have a major impact on the emotional state of this patient group (14).

The aim of the present study was to investigate patient-reported outcomes measures (PROMs) such as health related QoL, the prevalence of anxiety and depression in a group of patients with severe primary axillary hyperhidrosis (PAH), and the effects of microwave treatment for hyperhidrosis (MiraDry^®^) under real-life conditions. The study includes information on patient selection, evaluation of several different questionnaires related to QoL, and microwave treatment side effects over 1 year of follow-up. Severe PAH was defined as a score of 3 or 4 on the Hyperhidrosis Disease Severity Scale (HDSS).

## MATERIALS AND METHODS

### Data collection and subjects

Patients were recruited consecutively in 3 dermatology departments in Sweden: Vrinnevi Hospital in Norrköping, Linköping University Hospital, and Ryhov County Hospital in Jönköping, from September 2020 to June 2022, in compliance with the Declaration of Helsinki and International Council for Harmonization guideline for Good Clinical Practice.

Patients were required to have a PAH diagnosis according to the Swedish Hyperhidrosis Advisory Committee’s proposed criteria for the clinical diagnosis of primary focal hyperhidrosis (15) and an HDSS score of 3 or 4. The criteria are listed in **Table I**.

**Table I:** Criteria for clinical diagnosis of primary focal hyper-hidrosis (12)

Focal, visible, excessive sweating of at least 6 months’ duration without an identifiable cause with at least 2 of the following characteristics:
1. Bilateral and relatively symmetrical
2. Impairment of daily activities
3. Frequency of at least 1 episode per week
4. Age at onset of the disease under 25 years
5. Positive family history
6. Cessation of focal sweating during sleep

Individuals were excluded if they had had previous destructive treatment for PAH, botulinum toxin treatment for PAH in the last 6 months, allergic reaction to lidocaine, contraindication to epinephrine or treatment with antibiotics, previous infection or malignant/premalignant disease in or around the treatment area, previous diagnosis of hidradenitis suppurativa, pregnancy, breastfeeding, a pacemaker or other electronic implants, and finally if they were assessed to be unable or unlikely to attend the follow-up visits. Moreover, general patient information such as age, gender, weight, and height, as well as mental health conditions, was documented. The assigned Classification of Diseases version 10 diagnoses were obtained from the Swedish National Patient Register.

Evaluation of treatment results, side effects and local skin reactions was conducted through telephone calls and hospital visits. This was performed by a dermatologist using specific questions oriented towards side effects, in addition to validated instruments included in the study.

### Study design

Screening was performed before the start of the study, and only individuals who met the eligibility criteria were included in the study. Patients received their first treatment at baseline (Visit 1), and after 3 months (Visit 2) the effect of the treatment was assessed. The primary treatment endpoint was a reduction in HDSS to grade 2 or 1. Patients who still had an HDSS grade of 3 or 4 at Visit 2 were offered a second treatment. A final evaluation was performed 1 year after the first treatment (Visit 3). Secondary endpoints were changes in the Dermatology Life Quality Index (DLQI^©^), Hyperhidrosis Quality of Life Index (HidroQoL^©^), and Hospital Anxiety and Depression Scale (HADS^©^) from baseline to 3 months and to 1 year. In addition, participants were interviewed by telephone 2 weeks after each treatment to assess safety of treatment.

### Questionnaires/PROMs

Patients were asked to complete 4 questionnaires at Baseline (Visit 1), 3 months (Visit 2), and 1 year (Visit 3). Necessary licenses were obtained for the study.

HDSS consists of 4 statements concerning the impact of sweating on daily life, which can be scored from 1 to 4 points (15). The score increases according to the severity of the disease. An HDSS score of 1 corresponds to no or mild disease, 2 points means moderate disease, and 3 or 4 points constitute severe hyperhidrosis.

The DLQI consists of 10 statements, each rated between 0 and 3 points (16). It assesses how much a particular skin condition has affected a person’s life in the last week. The points are summarized (0–30), and the higher the final score, the more the disease has affected the patient’s quality of life. A Minimal Clinically Important Difference (MCID) score of 4 was suggested for the DLQI (17).

HidroQoL is a validated hyperhidrosis-specific questionnaire stating that it is a true QoL instrument (18, 19). Two impact domains are embedded within the HidroQoL; these are Daily Life Activities (6 items) and Psychosocial Life (12 items) domains. The higher the total score, the more severe the impact of hyperhidrosis is on the patient’s life. For HidroQoL, a score of 4 was suggested as MCID.

HADS is a questionnaire with 2 sections, 1 for anxiety and 1 for depression (20). Seven questions relate to anxiety (HADS-A), and the other 7 relate to depression (HADS-D). Scores for each scale range from 0 to 21, with scores categorized as follows: 0–7 is normal, 8–10 is borderline abnormal, and 11–21 is abnormal (21), although cut-off scores may vary depending on the study.

### Treatment protocol

Transcutaneous microwave ablation of sweat glands was performed using a device with a handpiece that emits microwaves (MiraDry^®^, Miramar Labs, Sunnyvale, CA, USA). The subjects in our study were treated according to the instructions for use by healthcare professionals who were certified and trained in the use of the MiraDry^®^ device.

Before the treatments, the area was anaesthetized with a tumescent solution (a standard mixture of carbocaine and epinephrine with normal saline) using a special multi-needle injector MESORAM^®^ (22). Special templates supplied with the device were used to outline the treated area. Treatment with the microwave device was started as soon as complete anaesthesia was achieved. Most patients were treated with the maximum energy level of 5 (however, in patients with very low body mass index or concave axilla, investigators chose a lower energy setting; this resulted in 16 female patients treated with energy level 4 and one patient with level 3).

### Statistical analysis

All PROMs were categorized as ordinal data, and a nonparametric test for paired data was applied (Wilcoxon’s signed-rank test), which was presented with medians. *P*-values < 0.05 were considered statistically significant. Statistical analysis was performed using IBM SPSS Statistics (Version: 27.0.0.0; IBM Corp, Armonk, NY, USA). The null hypothesis was the claim that 80% of the included patients would achieve an HDSS of 2 or 1 points at the end of the study. If the proportion that achieves a clinical effect (HDSS ≤ 2) is 90%, then 82 patients were needed to reject the null hypothesis with a power of at least 80% and a significance level of at most 5%. Given the dropout of patients who do not complete treatment or assessments (estimated at 20%), investigators intended to include 100 patients in the study.

## RESULTS

A total of 103 patients participated in the study: 56 patients from Norrköping study site (33 patients with an HDSS score of 3 and 23 patients with an HDSS score of 4); 38 patients from Linköping site (17 patients with HDSS 3 and 21 patients with HDSS 4), and 9 patients from Jönköping site (3 patients with HDSS 3 and 6 patients with HDSS 4). Most patients were women and were in their third decade of life (**Table II**). Eleven participants were lost to follow-up at Visit 2 and 18 at Visit 3. Among the 103 patients, 19 were taking psychotropic medications; 11 had previously been diagnosed with depression, 7 with an anxiety disorder, and 5 with attention deficit hyperactivity disorder.

**Table II:** Demographic features at baseline

Distribution of age	*n* (%)	*n* males (%)	*n* females (%)
18–24 years	22 (21.4)	5 (18.5)	17 (22.4)
25–34 years	44 (42.7)	13 (48.2)	31 (40.8)
35–44 years	28 (27.2)	9 (33.3)	19 (25)
45–65 years	9 (8.7)	0 (0)	9 (11.8)
Total years	103 (100)	27 (26.2)	76 (73.8)

### Hyperhidrosis Disease Severity Scale

**Fig. 1:**
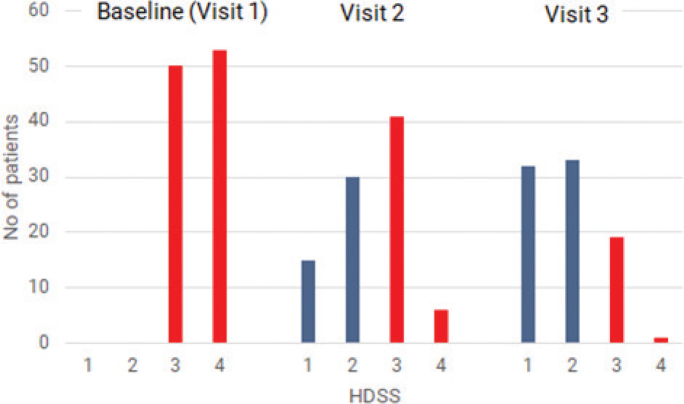
**Changes in the Hyperhidrosis Disease Severity Scale (HDSS) at baseline and follow-up visits.** HDSS scale: 1, sweating never noticeable and never interferes with daily activities; 2, sweating tolerable but sometimes interferes with daily activities; 3, sweating barely tolerable and frequently interferes with daily activities; 4, sweating intolerable and always interferes with daily activities. Visit 1, baseline; Visit 2, 3 months after the first microwave treatment which was performed at baseline; patients who still scored HDSS of 3 or 4 at Visit 2 would receive a second treatment; Visit 3, 1-year follow up from baseline.

Of the original 103 patients enrolled in the study, 45 underwent a second treatment based on the HDSS score at Visit 2. Two of the patients for whom a second treatment was indicated postponed the second treatment for non-medical reasons. Ten patients still achieved HDSS 3 or 4 despite 2 microwave treatments. In 11 patients who received only 1 microwave treatment, the HDSS score worsened from 2 or less (at 3 months) to 3 a year later. Overall, HDSS improved significantly after each visit (**Table III** and **Fig. 1**). No patient reported a higher HDSS score at the 1-year follow-up visit, compared with their score at baseline.

**Table III:** Outcome measures in patients with primary axillary hyperhidrosis at baseline and follow-up visits

Outcome measure	*n*	Median (IQR)	Improved compared with baseline *n* (%)	No change compared with baseline *n* (%)	Worsened compared with baseline *n* (%)	*p*-value
HDSSVisit1	103	3 (3; 4)				
HDSSVisit2	92	3 (2; 3)	66 (71.7)	26 (28.3)	0 (0.00)	< 0.001
HDSSVisit3	85	2 (1; 2)	75 (88.2)	10 (11.8)	0 (0.00)	< 0.001
HidroQolVisit1	103	26.00 (21.00; 31.00)				
HidroQolVisit2	91	16.00 (8.00; 26.00)	72 (79.1)	9 (9.9)	10 (11.0)	< 0.001
HidroQolVisit3	85	12.00 (4.00; 21.50)	79 (92.9)	2 (2.4)	4 (4.7)	< 0.001
DLQIVisit1	103	12.00 (8.00; 17.00)				
DLQIVisit2	92	6.00 (3.00; 11.75)	75 (81.5)	7 (7.6)	10 (10.9)	< 0.001
DLQIVisit3	85	4.00 (1.00; 10.00)	72 (84.7)	0 (0.0)	13 (15.3)	< 0.001
HADS-AVisit1	103	8.00 (5.00; 11.00)				
HADS-AVisit2	89	7.00 (4.00; 9.50)	52 (58.4)	6 (6.7)	31 (34.8)	0.001
HADS-AVisit3	85	6.00 (3.00; 9.00)	57 (67.1)	11 (12.9)	17 (20.0)	< 0.001
HADS-DVisit1	103	4.00 (1.00; 8.00)				
HADS-DVisit2	89	3.00 (1.00; 6.00)	41 (46.1)	22 (24.7)	26 (29.2)	0.056
HADS-DVisit3	85	2.00 (1.00; 4.50)	46 (54.1)	14 (16.4)	25 (29.4)	0.003

From baseline (Visit1) to 3 months (Visit 2) and to 1-year follow-up (Visit 3). First microwave treatment was performed at baseline; patients who still scored Hyperhidrosis Disease Severity Scale (HDSS) of 3 or 4 at Visit 2 would receive a second treatment.

IQR: interquartile range; DLQI, Dermatology Life Quality Index; HidroQoL, Hyperhidrosis Quality of Life Index; HADS, Hospital Anxiety and Depression Scale (A, Anxiety; D, Depression).

### Hyperhidrosis Quality of Life Index

**Fig. 2:**
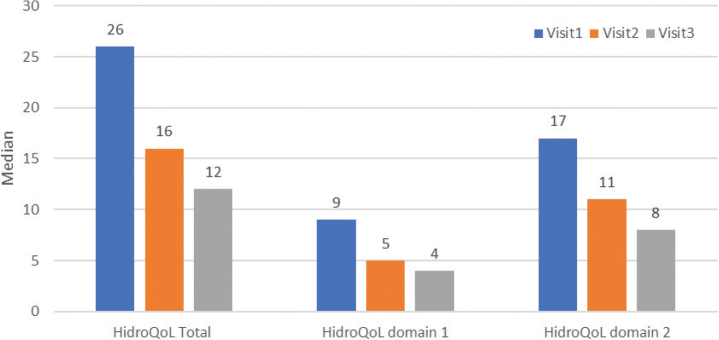
**Changes in the Hyperhidrosis Quality of Life Index (HidroQoL) at baseline and follow-up visits.** HidroQoL has 2 impact domains: domain 1, Daily Life Activities (6 items, ranges from 0 to 12) and domain 2, Psychosocial Life (12 items, ranges from 0 to 24). The total score is computed based on all 18 items, and ranges from 0 to 36. Visit 1, baseline; Visit 2, 3 months after the first microwave treatment which was performed at baseline; patients who still scored Hyperhidrosis Disease Severity Scale of 3 or 4 at Visit 2 would receive a second treatment; Visit 3, 1-year follow up from baseline. Improvement was statistically significant both at Visit 2 and Visit 3.

Change in the medians for HidroQoL is presented in Table III and **Fig. 2**. The HidroQoL total score improved significantly from a median of 26 at Visit 1 to 16 at Visit 2 (*p* < 0.001) and 12 at Visit 3 (*p* < 0.001).

### Dermatology Life Quality Index

Change in the medians for DLQI is presented in Table III. There was a significant improvement in DLQI scores from a median score of 12 at Visit 1 to 6 at Visit 2 (*p* < 0.001) and 4 at Visit 3 (*p* < 0.001).

### Hospital Anxiety and Depression Scale

**Fig. 3:**
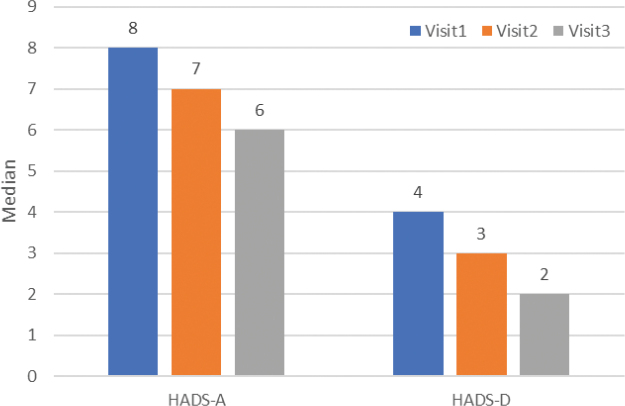
**Changes in the Hospital Anxiety and Depression Scale (HADS) at baseline and follow-up visits.** HADS (A, Anxiety; D, Depression). Visit 1, baseline; Visit 2, 3 months after the first microwave treatment which was performed at baseline; patients who still scored Hyperhidrosis Disease Severity Scale (HDSS) of 3 or 4 at Visit 2 would receive a second treatment; Visit 3, 1-year follow up from baseline. Improvement was statistically significant in HADS-A both at Visit 2 and Visit 3, and for HADS-D at Visit 3.

For the HADS, the 2 subscales HADS-A and HADS-D were analysed separately (see Table III and **Fig. 3**).

The median HADS-A score was 8 at Visit 1, 7 at Visit 2 (*p* = 0.001) and 6 at Visit 3 (*p* < 0.001). At Visit 1, 26 patients had a HADS-A score of 11 or higher, while only 7 patients had a score of 11 or higher at Visit 3.

The median HADS-D score at Visit 1 was 4, at Visit 2 it had dropped to 3 (*p* = 0.056) and at Visit 3 it was 2 (*p* = 0.003). Seven patients had a HADS-D score of 11 or higher at baseline.

### Side effects

Pain was a common adverse event after treatment (within the first 24–48 h), with 21 patients experiencing it as “very painful” (as described by the patients themselves during telephone follow-ups) and interfering with sleep. Two patients stated that they would not be willing to do the procedure 1 more time because of the pain. Post-treatment nodules were also commonly described, persisting for up to 3 months in 28 participants and were still present 1 year after treatment in 2 participants. Other less common side effects included oedema on adjacent arm or trunk (in 5 patients), tight band (in 4 patients), and 3 patients described an onset of sweating in other parts of the body that were not affected before treatment (face/trunk).

## DISCUSSION

The current study examined baseline parameters of QoL, anxiety, and depression in a cohort of patients with severe PAH. This was performed with instruments designed for evaluating health-related QoL at 3-month follow-up and 1-year follow-up after 1 and/or 2 microwave treatments compared with baseline. The results showed improvements in these parameters after each treatment, statistically significant 1 year after the first treatment.

A total of 88.2% achieved the primary endpoint of HDSS of 2 or less 1 year after 1 or 2 microwave treatments, in accordance with results from other studies (12, 23). One point of discussion is whether increasing treatment sessions would yield better and longer-lasting effects. Some studies did not find that increasing treatment sessions resulted in better efficacy (24) and could potentially lead to increased development of fibrosis in the treated area. Another method to improve treatment efficacy could have been to use a lower amount of anaesthetic solution with a different technique (25).

Anxiety and depression are the most common mental disorders, with depression being the leading cause of disability, according to the World Health Organization (26, 27). Hyperhidrosis is known to cause psychological stress, increased anxiety, and depression in patients with primary hyperhidrosis. Bragança et al. (28) described anxiety as prevalent in 49.6% among 197 patients with severe primary hyperhidrosis, whilst depression frequency was within the normal range compared to what is described in the general population. In 2016, Bahar et al. (29) published a study of patients from Canada and China in which the prevalence of anxiety and depression was found to be 21.3% and 27.2% in patients diagnosed with hyperhidrosis, respectively, compared with 7.5% with anxiety and 9.7% with depression in the control group. Kristensen et al. (30) showed that almost 50% of Swedish hyperhidrosis patients suffered from anxiety, while depression was within the normal range. Moreover, another study from Sweden showed improvement in HADS scores following botulinum toxin treatment, in a short-term survey (31). However, other studies argue against the common clinical model according to which hyperhidrosis is an expression of psychological distress (32). In our study, we showed a reduction in both HADS-A and HADS-D scores consistently over a year (although only marginally significant in the case of depression). This provides evidence showing that there is a strong relation between hyperhidrosis and psychological well-being.

Currently, there is increasing evidence that people with skin symptoms often suffer from social anxiety. Schut et al. (33) investigated the symptoms of body dysmorphic disorder in patients with dermatological conditions and found that patients with hyperhidrosis (a skin condition without obvious skin lesions) were the group most likely to have symptoms of body dysmorphic disorder. Our patients described that their sweating problem severely interfered with daily life, which we could not verify by optical assessment at the hospital visit (as it is difficult to measure real-time hyperhidrosis objectively in clinical practice). 11.8% still have high HDSS scores of 3 or 4 after 2 microwave treatments, and it would be interesting to investigate this particular patient group further. Could “impostor phenomenon” be one explanation in this group of patients (34)?

### Limitations

This study focused on PROMs, which provide a holistic approach to treatment assessment. There is always a risk of recall error when answering questionnaires, although the questionnaires used in this study asked patients to recall only the last week when answering the questions. The results reported by patients are subjective, and a person’s experience can be influenced by many things outside the control of the study. Additionally, the study was conducted during the COVID-19 pandemic. However, as regulations in Sweden did not change the standard of care during this period, we believe that our findings will facilitate knowledge in this field of research.

According to a newly published study that analysed safety reports associated with Miradry^®^ using the FDA Manufacturer and User Facility Device Experience (MAUDE) database, infections are the most prevalent side effects (35), though we did not register a single skin infection during our study. This might be explained by the fact that our study was performed under rigorous hygiene rules. Additionally, our study nurses were thoroughly trained to be able to provide the best of care to our patients.

Another limitation of the study is the short follow-up period (1 year). Further studies with a longer follow-up interval of up to 3 years are in progress.

### Conclusion

The patients enrolled in this study suffered from a severe form of PAH, with elevated scores of measures for health-related QoL and significant anxiety symptoms, thus highlighting the burden of excessive sweating in daily and psychosocial life. Treatment with a microwave device significantly improved hyperhidrosis, diminishing anxiety and depression symptoms, at an already difficult time (COVID-19 pandemic period). Although microwave therapy for hyperhidrosis is a painful treatment, almost all patients chose to undergo this procedure a second time to achieve greater improvement in their condition. This alternative treatment should be presented to patients (along with the risk of side effects) so that they can make an informed decision that could improve their quality of life over a longer period.
